# Effects in Short and Long Term of Global Postural Reeducation (GPR) on Chronic Low Back Pain: A Controlled Study with One-Year Follow-Up

**DOI:** 10.1155/2015/271436

**Published:** 2015-04-06

**Authors:** Chiara Castagnoli, Francesca Cecchi, Antonio Del Canto, Anita Paperini, Roberta Boni, Guido Pasquini, Federica Vannetti, Claudio Macchi

**Affiliations:** ^1^Fondazione Don Carlo Gnocchi, IRCCS, Scientific Institute, 50143 Florence, Italy; ^2^Department of Medical and Surgical Critical Area, Università degli Studi di Firenze, Florence, Italy

## Abstract

*Objective.* Comparing global postural reeducation (GPR) to a standard physiotherapy treatment (PT) based on active exercises, stretching, and massaging for improving pain and function in chronic low back pain (CLBP) patients. *Design.* Prospective controlled study. *Setting.* Outpatient rehabilitation facility. *Participants.* Adult patients with diagnosis of nonspecific, chronic (>6 months) low back pain. *Interventions*. Both treatments consisted of 15 sessions of one hour each, twice a week including patient education. *Measures.* Roland Morris Disability Questionnaire to evaluate disability, and Numeric Analog Scale for pain. A score change >30% was considered clinically significant. Past treatments, use of medications, smoking habits, height, weight, profession, and physical activity were also recorded on baseline, on discharge, and 1 year after discharge (resp., T0, T1, and T2). *Results.* At T0 103 patients with cLBP (51 cases and 52 controls) were recruited. The treatment (T1) has been completed by 79 (T1) of which 60 then carried out the 1-year follow-up (T2). Both GPR and PT at T1 were associated with a significant statistical and clinical improvement in pain and function, compared to T0. At T2, only pain in GPR still registered a statistically significant improvement.

## 1. Introduction

Pain is an unpleasant sensory and emotional experience associated with actual or potential tissue damage [1, 2]. Pain becomes chronic when it persists longer than the expected period of healing [1, 3], that is, 3 months [3, 4] In chronic pain, the sensorial process becomes abnormal, leading to detectable changes in central nervous system data processing, motor control, and the experience of pain itself [2, 5]. Low back pain (LBP) is defined as pain and/or discomfort located below the costal margin and above the inferior gluteus folds, with or without related leg pain [6]. Approximately 70–85% of individuals will experience LBP during their lifetime, and over 80% of them will report recurrent episodes. It is estimated that 80–90% of subjects would recover within 6 weeks, regardless of the type of treatment; however, 5–15% will develop chronic LBP [7]. Low back pain has a relevant impact on patients in terms of pain, activity limitations, participation restrictions, influence on career, use of sanitary resources, and financial burdens [6, 7].

The treatment of chronic LBP is still very controversial. International guidelines consider three groups of low back pain treatment options: medication, invasive, and conservative treatment. The conservative approach is generally recommended for chronic nonspecific low back pain: patient education, specific exercise, and spinal manipulation are claimed to be effective in the short term, but the magnitude of functional improvement and pain relief is generally low. Stretching, spine stabilization, and proprioception exercise are generally recommended with or without manual therapy or massage. According to a recent Cochrane review [8], exercise therapy has evidence of being effective in the treatment of chronic nonspecific low back pain in the short and long term, especially individual exercise programs carried out under the supervision of a physiotherapist. It was also seen that stretching exercises and muscle strengthening are those which combined with stabilization exercises of the trunk are associated with the best results in terms of pain reduction and the recovery of function. Individual-based physiotherapy exercise protocols for chronic low back pain are widely used, even if the cost-benefit ratio is now still under discussion [9].

In the rehabilitative treatment of low back pain many approaches are used around the world, requiring specifically trained physiotherapists but claiming long lasting benefits. Global postural reeducation (GPR) is an approach based on an integrated idea of the muscular system as formed by muscle chains, which can face shortening resulting from constitutional, behavioral, and psychological factors. The aim of GPR is to stretch the shortened muscles using the creep property of viscoelastic tissue and to enhance the contraction of the antagonist muscles [4, 10]. The GPR assessment investigates the role and status of the so-called “muscle chains,” the functional groups of muscles responsible for posture and its alterations. It also analyzes the extent to which muscle chains influence each other and how their alterations can accumulate in each subject and whether and to what extent these alterations are responsible for the symptoms reported. This is a method that is not currently part of university teaching and requires a specific training for physiotherapists in private schools after graduation; the basic course lasts four weeks distributed over a year followed by several courses of superior training.

The scientific literature regarding the use of GPR for the treatment of chronic low back pain is scarce. A recent review of the literature identified 11 studies [11] concerning scientific evidence of the effectiveness of GPR in the treatment of various conditions. Four of these studies were randomized controlled trials. Important results were reported by Moreno et al. [12] about the effect of GPR on respiratory capacity, Cabral et al. [13] about positive effects on the patellofemoral syndrome, and Fernandez de-la-pena in 2005 about ankylosing spondylithis [14]. However only one study [2] investigates the effects of GPR on neck pain and no studies were considered for LBP.

Our pragmatic clinical study aims at describing the short and long term effects of GPR on patients with nonspecific cLBP, compared with a similar sample of control that received standard individual physiotherapy (PT).

## 2. Subjects and Methods

### 2.1. Oversight

All patients signed their informed consent form to be included in the study. The Institutional Review Board approved the study protocol.

### 2.2. Participants

All patients at the Don Gnocchi Foundation IRCCS Florence from June 2011 to February 2011 with a prescription of individual PT for persisting nonspecific low back pain were considered eligible. Diagnosis and prescription were given by community health specialists in physical medicine and rehabilitation: some specify the type of individual PT prescription (e.g., GPR, McKenzie etc.) while others just provide a generic prescription of individual PT, leaving the choice of approach to the physiotherapist. For the purpose of our study, further inclusion criteria were 18–80 year old patients, and chronic LBP, defined as persisting from “very often” to “always” for at least 6 months. Exclusion criteria were neurological signs (irritation/deficit) and/or pain below the kneecap; severe osteoporosis; spondylolisis and spondylolisthesis; arthritis (rheumatoid arthritis, spondylitis, etc.); tumors; infections; previous spinal surgery; other debilitating and/or very painful musculoskeletal conditions; recent trauma (<30 days); acute illness; anticoagulant therapy or phenobarbital or radio/chemotherapy; psychiatric illness; and medical-legal disputes in progress; pregnancy. Eligible patients meeting the above criteria were invited to participate in the study and were asked for their written consent. The Institutional Review Board of the Don Gnocchi Foundation approved the study protocol. The patients who already had a specific prescription of individual PT-GPR were assigned to the GPR group, while those with a general prescription of individual PT and no contraindications to GPR (assessed by a specialist in physical medicine and rehabilitation) were either addressed to GPR or to PT to form a matched control group. Six qualified physiotherapists, with basic training and two courses of higher education in accordance with GPR Souchard and with at least 5-year experience, assessed and delivered the rehabilitation program, delivering either GPR or PT according to group assignment.

### 2.3. Intervention

For both interventions the program provided 15 sessions of 60 minutes each, two times a week. Therapist assessment was individually performed the day before the start of the treatment, while the final assessment was delivered immediately after the end of the last session. The exercise treatment protocol (PT) was formulated according to national and international guidelines on the treatment of persistent low back pain with exercises [10, 15] and in accordance with the Tuscany Region resolution. Exercise was focused on stimulating awareness of the body scheme, balancing muscle function (decontraction of the shortened muscles, strengthening of weakened muscles), stabilizing the spine, and correcting any alteration of postural alignment. Each treatment was individualized for every patient and for his/her pain-related limitation. The physiotherapists chose the most appropriate exercises from the standardized protocol.

In GPR group patients were subjected to a postural assessment according to the Souchard approach. Different body segments were observed in relation to patients and space, in order to identify possible disharmonies. Patient assessment is global and takes into account any changes in vision, dental occlusion, the support of the foot, visceral or psychological problems, past neurological or orthopedic problems, and a search for shortened muscle chains. According to specific assessment, appropriate “postures” were selected to correct identified muscle imbalances [16–18].

At the beginning of the treatment, each patient, in both groups, received an informative brochure with evidence based standardized educational information on basic back anatomy and biomechanics, optimal postures, correct movements in daily living activities, and ergonomics. On discharge each patient received an individual short set of exercise programs to carry out at home. These were generally recommended as well as the regular practice of low-impact physical activity of low to moderate intensity, according to the clinical profile and preferences of the patients.

### 2.4. Measures

Measures were taken at T0 (baseline), T1 (discharge, 15 working days from baseline), and T2 (twelve months from discharge). Baseline assessment was of general characteristics: age, sex, BMI, smoking, number of years or months of low back pain suffering, pain frequency in the last 6 months, pain related to the use of drugs, whether previous LBP treatments performed also with different approach (massage, physical therapy, acupuncture, etc.), profession, number of work days lost due to back pain, and physical activity. The primary outcome measure was low back pain-related functional disability, assessed by the Roland and Morris Disability Questionnaire (RMDQ) [8].

The Roland Morris score ranges from 0 to 24 ranging, respectively, from “zero” to “maximum” low back pain-related disability. We regarded as “respondent” patients with a minimal clinically important difference (MCID) in scores from the Roland Morris Disability Questionnaire (RMDQ), indicated by the literature as an improvement equal to or greater than 30% compared to baseline at both ends of the treatment and follow-up [9]. In addition, the NRS (Numeric Rating Scale) consists of a line numbered from 0 to 10 that represents pain severity levels from “none” to “most intense pain imaginable” [19].

Afterwards, between December 2012 and February 2013 (T2), all the patients who completed the treatment were contacted for a follow-up interview, performed by an independent researcher, reassessing the RMDQ and NRS and enquiring about any medication or other treatment for CLBP received, about the practice of any regular physical activity, any changes in activity or profession in the previous year, and any adherence to a specific exercise program.

### 2.5. Statistical Analysis

The sample sizes were calculated with a priori sample size analysis. From data of previous studies [20, 21] hypothesizing an anticipated effect size (Cohen's d) of 0.8 and a statistical power of 0.8 and a probability level of 0.05 the minimum sample size per group (two-tailed hypothesis) results in being 26 subjects. So we can retain our sample (30 subjects per group) as being appropriate.

Statistical analysis was performed using the software STATA 7.0, from Stata Corporation (College Station, Texas, USA).

For analyzing the differences between the two groups we used

- for continuous variables such as age, weight, height, the Student's *t*-test for independent sample.
- For categorical variables such as sex, smoke yes/no, use of drugs we used the Pearson *χ* ^2^ test.

For analyzing the differences concerning the score of disability and pain before/after within the same group we used the Wilcoxon sign rank test.

For analyzing the differences between groups concerning the score of disability and pain we used the Kruskal-Wallis rank test and Pearson *χ* ^2^ test.

In the text (Tables 2-3) the score of disability (Roland Morris score) and pain (NRS score) were reported as mean and standard deviation but the analyses of these variables were conducted appropriately with rank tests as mentioned above.

## 3. Results

**Figure 1:**
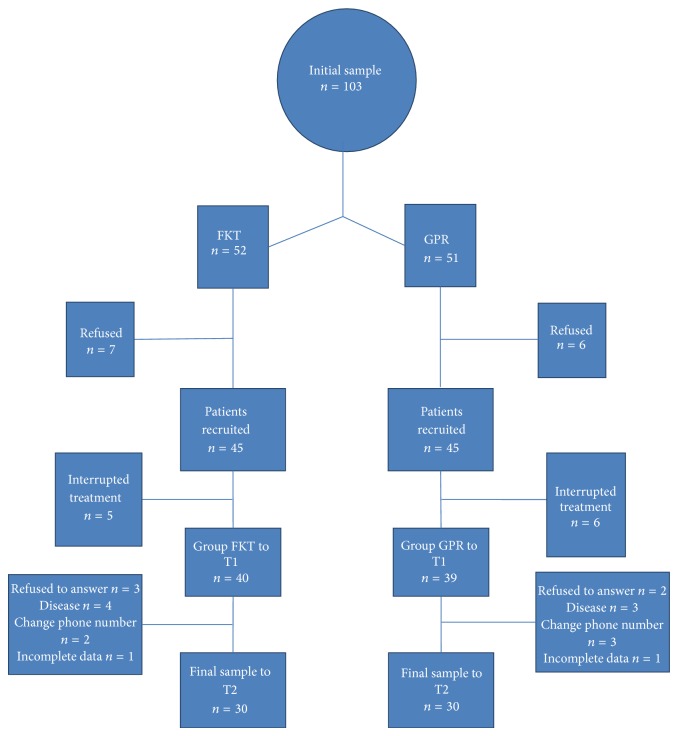
Study flowchart.

From June 2011 to February 2011, 103 patients diagnosed with persistent chronic low back pain attended our facility for rehabilitation treatment. 32 of them had a specific prescription of GPR, while 71 had a generic prescription of individual PT. All the patients were assessed as eligible with no contraindications for GPR and all of them met the inclusion criteria. Those with GPR prescription were assigned to GPR, while the others were alternatively assigned to GPR or to PT to form a paired control group. Of the 52 patients assigned to GPR and the 51 to PT only 79 patients (39 for the GPR group and 40 for the PT group) were part of the final sample at T1 because 13 eligible patients refused to participate in the study (7 for the FKT group and 6 for the GPR group) and 11 patients discontinued the treatment (5 for the PT group and 6 for the GPR group). Of the 79 patients recruited 19 did not complete the 1 year follow-up. The final sample to T2 was composed of 60 persons, 30 for each group (Figure 1).

Demographic, clinical, and general characteristics of the two groups at baseline (T0) are summarized in Table 1.

No statistically significant differences were observed between the two groups in the examined variables. The distribution of the two sexes in the two groups was homogeneous. The two groups were not homogeneous regarding profession; in the GPR group, in fact, employees are 50% (=15 subjects) while in the PT group only 8 patients (26%) were employed.

Therefore, in both groups and for both outcomes there was a statistically significant improvement in RMDQ and NRS scores on discharge (*P* < 0,001). Improvement equal or above 30% was considered a clinically significant difference, as indicated in literature both for NRS and for RMDQ scores [22]. We thus classified those who improved their RMDQ score by at least 30% as responders. For both outcomes, we found a greater number of responders in the GPR group compared to the PT group, although the difference was not statistically significant. At follow-up, 1 year after discharge (T2) we found an improvement in both NRS and RMDQ compared to T0, but only pain relief, expressed by NRS improvement, was statistically but not clinically significant in the GPR group (*P* < 0.02). Comparing responders in the two groups at T2, we found that their percentage was not significantly different between groups, either for NRS (*P* < 1.00) or RMDQ (*P* < 0.27) (Tables 2 and 3).

Table 4 shows the comparison between the two groups of the variables collected by the structured follow-up questionnaire (T2).

We found a statistically significant difference only for the time elapsed before receiving further treatment in follow-up. (*P* < 0.02): In fact, during the year following the end of treatment, GPR patients reported that they were subjected to further physiotherapy treatments later in time than patients in the PT group.

## 4. Discussion

The findings of this prospective controlled study on patients with cLBP show that GPR patients reported similar improvement in pain and function as those who received standard physical therapy in the short term, as both treatments were associated with statistically significant improvements in function and pain, while only GPR treatment was associated with statistically significant pain relief at the one-year follow-up.

In the literature [19, 23, 24] a RMDQ score difference from 2,5 to 6,8 in low initial scores (less than 15) and from 5,5 to 13,8 in high initial scores (more than 15) is considered clinically significant; this is greater than the normally anticipated score considering the natural history of the disorder. As to the NRS score, a score reduction of at least 30% is considered as the minimal important clinical difference (MICD) [22]. Thus, we can conclude that, in our sample, both treatments under consideration were associated with clinically significant improvements in related pain and disability in the short term. This result differs from a recent Italian nonrandomized trial comparing GPR with stabilization exercises in persistent low back pain [3] which showed a greater improvement in the GPR group in outcomes in short and middle terms (3–6 months). While another study (the randomized controlled trial of GPR in the treatment of mechanic back pain) concerning neck pain [2] provided results more similar to ours: in this study GPR is compared with traditional stretching in a sample of women (31); both groups showed significant pain relief and a range of motion improvement results following the treatment and a small reduction at follow-up time. At follow-up (six weeks after the end of the treatment) there was improvement in all domains, except that both groups reported increased pain. There were no significant differences between groups.

In the long term (1 year follow-up) we found an improvement in both NRS and RMDQ scores compared to T0 in both groups, but neither of the two scores was clinically significant. In our findings, discharge improvements were attenuated in time and were no more clinically significant in either group; however patients who received GPR still reported statistically significant pain relief compared to baseline and a lower frequency of pain which may indicate a longer-lasting effect of such treatment on pain.

However, since we have evaluated patients not earlier than one year after discharge, we can not say if significant improvements were maintained longer in RPG because we could not assess whether improvements have been lost more in the first than in the second half after the end of treatment, as it suggests a recent study LBP. [19].

In a randomized clinical trial in 2008, conventional stretching and muscle chain stretching in association with manual therapy were equally effective in reducing pain and improving the range of motion and quality of life of female patients with chronic neck pain, immediately after treatment and the results were maintained at a six-week follow-up, differing from our sample [2]. This suggests the need for a continuous exercise program; we did not anticipate this in our protocol, but it could be included in the future to enable patients to maintain the positive results obtained from the treatment.

The main limitation of our study was the assignment of patients to the two groups that were not possible to randomize. Although all our patients had a clinical indication for GPR, only those who had a general prescription could be casually assigned to either group, while the few that already came to our facility with a prescription of GPR were necessarily assigned to the GPR group. Although we verified that the two groups were similar for all relevant general and clinical characteristics, our findings do not have the strength of a randomized controlled study.

LBP shows complex and variable clinical features. Further, in future studies, in order to give more solid proof to our thesis it would be interesting to divide the sample into subgroups with similar characteristics at clinical assessment and to subject the patients to single interventions, comparing these results to a control group [20, 25]. In particular, after GPR assessment, the choice to divide the sample into subgroups according to similar patterns of muscle retraction would allow a more focused comparison [18].

Since treatments provided similar clinically significant improvements in both pain and disability, without significant differences between groups, we can say that the two approaches were equivalent for our cLBP patients.

## 5. Conclusions

This study compared the short and long term effects of GPR and individual PT on cLBP. Our result showed equivalent improvements both in function and pain: both outcomes presented short term improvements above the clinically minimal significant difference that were no more clinically significant at one year. Only in the GPR group was pain relief statistically significant at one year in frequency and intensity. Considering that GPR is more expensive in terms of the professional training by physiotherapists, our results do not recommend promoting its systematic application in cLBP. Nevertheless, our data suggest the possibility that GPR may have longer lasting effects compared to PT, which deserves further investigation by a randomized controlled trial.

## Figures and Tables

**Table 1:** Patients Characteristics at baseline.

	GPR Group		FKT Group		Significativity (*P*)
General characteristics					
Sex (*n*) (m/f)	4/26		7/23		0,317^†^
Age (years) (mean) (DS)	58,97	0,44	62,54	13,19	0,501^*^
Smoke (*n*)	Yes-13/30		Yes-17/30		0,278^†^
Weight (Kg) (mean) (DS)	63,63	9,29	67,60	12,43	0,160^*^
Height (cm) (mean) (DS)	162,90	6,27	163,70	10,28	0,717^*^
Clinical characteristics					
Pain duration, years (mean) (DS)	15,01	13,20	10,93	12,97	0,240^*^
Frequency of pain (*n*)					
(i) Quite often	8		10		0,176^†^
(ii) Very often	6		11		
(iii) Always	16		9		
Use of drugs (*n*)	Yes-24/30		Yes-20/30		0,121^†^
Frequency of drugs (*n*)					
(i) Little	7		9		0,629^†^
(ii) Enough	5		6		
(iii) Often	1		4		
(iv) Always	0		1		
Previous treatments (*n*)	Yes-6/30		Yes-3/30		0,053^†^
Kind of job (*n*)	Yes-26/30		Yes-22/30		0,277^†^
Number of lost working days (mean) (DS)	0,36	0,49	0,35	0,49	0,930^*^
Physical activity (*n*)	11		12		0,871^†^

^∗^Student *t*-test for independent samples.

^†^Pearson *χ* ^2^ test.

**Table 2:** Differences in NRS results per group before, after and between groups.

	NRS T0 Mean ± SD	NRS T1 Mean ± SD	Responders NRS T1 (*n*)	NRS T2 Mean ± SD	Responders NRS T2 (*n*)	Significativity NRS T0 versus T1 (*P*)	Significativity NRS T0 versus T2 (*P*)
GPR	6,7 ± 2,28	3,73 ± 2,68	21/30	5,73 ± 4,38	8/30	<0,001^*^	0,02^*^
FKT	7,2 ± 2,25	4,43 ± 2,35	16/30	6,5 ± 2,03	8/30	<0,001^*^	0,12^*^
GPR versus FKT (*P*)	0,3^†^	0,15^†^	0,18^††^	0,23^†^	1^††^		

^∗^Wilcoxon sign test.

^†^Kruskal-Wallis rank test.

^††^Pearson *χ* ^2^ test.

**Table 3:** Differences in RMDQ results per group before, after and between groups.

	RMDQ T0 Mean ± SD	RMDQ T1 Mean ± SD	Responders RMDQ T1 (*n*)	RMDQ T2 Mean ± SD	Responders RMDQ T2 (*n*)	Significativity RMDQ T0 versus T1 (*P*)	Significativity RMDQ T0 versus T2 (*P*)
GPR	10,97 ± 4,38	5,1 ± 4,51	26/30	9,67 ± 6,13	12/30	<0,001^*^	0,24^*^
FKT	12,47 ± 5,45	6,43 ± 5,03	24/30	11,2 ± 6,29	8/30	<0,001^*^	0,12^*^
GPR versus FKT (*P*)	0,21^†^	0,27^†^	0,48^††^	0,36^†^	0,27^††^		

^∗^Wilcoxon sign test.

^†^Kruskal-Wallis rank test.

^††^Pearson *χ* ^2^ test.

**Table 4:** Comparison of variables of low back pain questionnaire at T2.

	GPR group		FKT group		Significativity (*P*)
Smoke (*n*)	Yes-5/30		Yes-5/30		1,000^†^
Years from treatment (mean) (DS)	1,61	0,48	1,59	0,45	0,891^*^
Pain frequency (*n*)					
(i) Quite often	16		7		0,048^†^
(ii) Very often	7		10		
(iii) Always	7		13		
Previous treatment (*n*)	Yes-15/30		Yes-11/30		0,302^†^
Kind of job (*n*)					
(i) Employees	16		6		0,061^†^
(ii) Autonomous	0		2		
(iii) Housewife	12		17		
(iv) Doesn't work	2		5		
Working days lost (*n*)	Yes-15/17		Yes-8/9		0,561^†^
Physical activity (*n*)	Yes-11/30		Yes-16/30		0,213^†^
Use of drugs (*n*)	Yes-14/30		Yes-15/30		0,796^†^
Frequency of drugs (*n*)					
(i) Little	7/14		4/15		0,796^†^
(ii) Enough	6/14		4/15		
(iii) Often	0/14		2/15		
(iv) Always	1/14		5/15		

^∗^Student *t*-test for independent samples.

^†^Pearson *χ* ^2^ test.

## List of Abbreviations

LBP: Low back pain
CLBP: Chronic low back pain
GPR: Global postural reeducation
PT: Protocol treatment
MCID: Minimal clinically important difference
RMDQ: Roland and Morris Disability Questionnaire
NRS: Numeric Rating Scale
BMI: Body Mass Index.

## References

[1] (1986). Classification of chronic pain. Descriptions of chronic pain syndromes and definitions of pain terms. *Pain Supplements*.

[2] Cunha A. C. V., Burke T. N., França F. J. R., Marques A. P. (2008). Effect of global posture reeducation and of static stretching on pain, range of motion, and quality of life in women with chronic neck pain: a randomized clinical trial. *Clinics*.

[3] Bonetti F., Curti S., Mattioli S. (2010). Effectiveness of a “Global Postural Reeducation” program for persistent Low Back Pain: a non-randomized controlled trial. *BMC Musculoskeletal Disorders*.

[4] Andersson H. I. (1994). The epidemiology of chronic pain in a Swedish rural area. *Quality of Life Research*.

[5] Farina S., Tinazzi M., Le Pera D., Valeriani M. (2003). Pain-related modulation of the human motor cortex. *Neurological Research*.

[6] Airaksinen O., Brox J. I., Cedraschi C. (2006). Chapter 4: European guidelines for the management of chronic nonspecific low back pain. *European Spine Journal*.

[7] Liddle S. D., Baxter G. D., Gracey J. H. (2004). Exercise and chronic low back pain: what works?. *Pain*.

[8] Hayden J. A., van Tulder M. W., Tomlinson G. (2005). Systematic review: strategies for using exercise therapy to improve outcomes in chronic low back pain. *Annals of Internal Medicine*.

[9] Kääpä E. H., Frantsi K., Sarna S., Malmivaara A. (2006). Multidisciplinary group rehabilitation Versus individual physiotherapy for chronic nonspecific low back pain: a randomized trial. *Spine*.

[10] Costa L. O., Maher C. G., Latimer J. (2009). Motor control exercise for chronic low back pain: a randomized placebo-controlled trial. *Physical Therapy*.

[11] Teodori R. M., Negri J. R., Cruz M. C., Marques A. P. (2011). Global postural re-education: a literature review. *Revista Brasileira de Fisioterapia*.

[12] Moreno M. A., Catai A. M., Teodori R. M., Borges B. L. A., de Cesar M. C., da Silva E. (2007). Effect of a muscle stretching program using the Global Postural Re-education method on respiratory muscle strength and thoracoabdominal mobility of sedentary young males. *Jornal Brasileiro de Pneumologia*.

[13] Cabral C. M. N., Yumi C., de Sacco I. C. N. (2007). Eficácia de duas técnicas de alongamento muscular no tratamento da síndrome femoropatelar: um estudo comparativo. *Fisioterapia e Pesquisa*.

[14] Fernández-De-Las-Peñas C., Alonso-Blanco C., Morales-Cabezas M., Miangolarra-Page J. C. (2005). Two exercise interventions for the management of patients with ankylosing spondylitis: a randomized controlled trial. *American Journal of Physical Medicine and Rehabilitation*.

[15] Hayden J. A., van Tulder M. W., Malmivaara A., Koes B. W. (2005). Exercise therapy for treatment of non-specific low back pain. *Cochrane Database of Systematic Reviews*.

[16] PhE S. (1994). *Basi del Metodo di Rieducazione Posturale Globale-Il Campo Chiuso*.

[17] Souchard P.-E., Meli O., Sgamma D., Pillastrini P. (2009). Rieducazione posturale globale. *EMC-Medicina Riabilitativa*.

[18] Souchard P., Ollier M., Zani C., Sgamma D. (2002). *Le Scoliosi: Trattamento Fisioterapico e Ortopedico*.

[19] Kovacs F. M., Abraira V., Royuela A. (2007). Minimal clinically important change for pain intensity and disability in patients with nonspecific low back pain. *Spine*.

[20] Cecchi F., Molino-Lova R., Chiti M. (2010). Spinal manipulation compared with back school and with individually delivered physiotherapy for the treatment of chronic low back pain: a randomized trial with one-year follow-up. *Clinical Rehabilitation*.

[21] Cecchi F., Pasquini G., Paperini A. (2014). Predictors of response to exercise therapy for chronic low back pain: result of a prospective study with one year follow-up. *European Journal of Physical and Rehabilitation Medicine*.

[22] Ostelo R. W. J. G., Deyo R. A., Stratford P. (2008). Interpreting change scores for pain and functional status in low back pain: towards international consensus regarding minimal important change. *Spine*.

[23] Heymans M. W., van Tulder M. W., Esmail R., Bombardier C., Koes B. W. (2005). Back schools for nonspecific low back pain: a systematic review within the framework of the Cochrane Collaboration Back Review Group. *Spine*.

[24] Stratford P. W., Binkley J., Solomon P., Finch E., Gill C., Moreland J. (1996). Defining the minimum level of detectable change for the Roland-Morris questionnaire. *Physical Therapy*.

[25] Cleland J. A., Frit J. M., Childs J. D., Kulig K. (2006). Comparison of the effectiveness of three manual physical therapy techniques in a subgroup of patients with low back pain who satisfy a clinical prediction rule: study protocol of a randomized clinical trial [NCT00257998]. *BMC Musculoskeletal Disorders*.

